# Folate intake, serum folate levels and esophageal cancer risk: an overall and dose-response meta-analysis

**DOI:** 10.18632/oncotarget.14432

**Published:** 2017-01-02

**Authors:** Yan Zhao, Chenyang Guo, Hongtao Hu, Lin Zheng, Junli Ma, Li Jiang, Erjiang Zhao, Hailiang Li

**Affiliations:** ^1^ Department of Radiology Intervention, Affiliated Tumor Hospital of Zhengzhou University, Zhengzhou, Henan, China; ^2^ Department of Epidemiology and Biostatistics, Affiliated Tumor Hospital of Zhengzhou University, Zhengzhou, Henan, China

**Keywords:** folate, esophageal cancer, dose-response, meta-analysis

## Abstract

Previously reported findings on the association between folate intake or serum folate levels and esophageal cancer risk have been inconsistent. This study aims to summarize the evidence regarding these relationships using a dose-response meta-analysis approach. We performed electronic searches of the Pubmed, Medline and Cochrane Library electronic databases to identify studies examining the effect of folate on the risk of esophageal cancer. Ultimately, 19 studies were included in the meta-analysis. Summary odds ratios (ORs) were estimated using a random effects model. A linear regression analysis of the natural logarithm of the OR was carried out to assess the possible dose-response relationship between folate intake and esophageal cancer risk. The pooled ORs for esophageal cancer in the highest vs. lowest levels of dietary folate intake and serum folate were 0.63 (95% CI: 0.56-0.71) and 0.71 (95% CI: 0.55-0.92), respectively. The dose-response meta-analysis indicated that a 100 μg/day increment in dietary folate intake reduced the estimate risk of esophageal cancer by 12%. These findings suggest that dietary and serum folate exert a protective effect against esophageal carcinogenesis.

## INTRODUCTION

Esophageal cancer is the most common upper gastrointestinal malignant tumor, ranking as the sixth most common cancer and the eighth leading cause of cancer deaths in the world [1]. At present, most esophageal cancers are detected when the disease is already in an advanced stage and this is reflected in the 5-year survival rate being less than 20% [2]. Therefore, identifying modifiable risk factors and developing primary prevention programs are of paramount importance.

Diet is an important modifiable factor that can modulate carcinogenesis. Among the dietary supplemental vitamins, folate shows promise in reducing the risk of carcinogenesis at some doses. Folate is a water-soluble B vitamin found in citrus fruits, green leafy vegetables, cruciferous vegetables and legumes, among others [3]. Folate deficiency can promote carcinogenesis by stimulating aberrant DNA methylation resulting in defective activation of oncogenes [4]. Low folate levels reduce *de novo* thymidylate biosynthesis that will induce uracil mis-incorporation during DNA repair and synthesis resulting in DNA mutagenesis that in addition to DNA strand breaks and chromosomal damage triggers malignant transformation [5]. Although many epidemiologic studies have shown that low folate levels increase the risk of human cancers, the role of folate intake in esophageal cancers has remained controversial [6–8]. Therefore, to characterize the link between folate levels and the risk of esophageal cancer, and to evaluate the dose-response relationship of esophageal cancer and folate intake, we performed a meta-analysis of the current epidemiological literature.

## RESULTS

### Literature search

**Figure 1:**
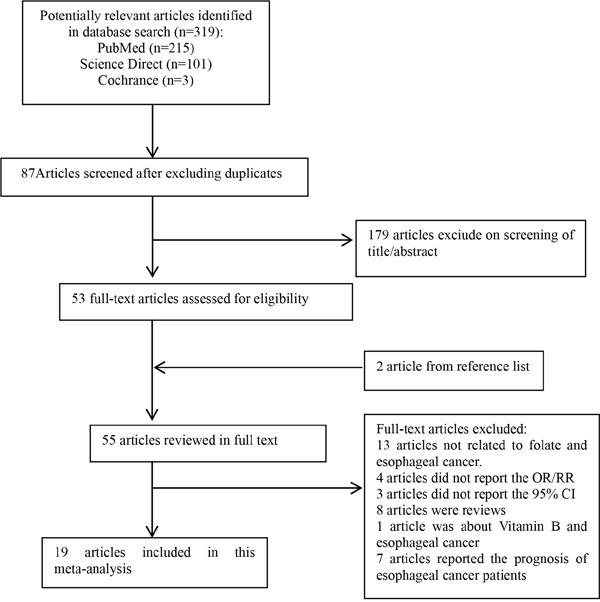
The flow diagram of screened, excluded and analyzed publications

Figure 1 shows the search results and literature selection for this study. Of the 319 articles we initially identified from PubMed, Medline and Cochrance Library, 87 were eliminated as they reported on the same population data. Then, after reviewing the title and abstract of the remaining 232 articles, 179 were excluded as irrelevant. On the other hand, 2 relevant articles were added after a manual search of the reference lists. The full texts of the remaining 55 articles were reviewed to eliminate those (1) that were reviews (2) that did not report the association between folate and risk of esophageal cancer (3) that did not report the OR/RR/ 95% CI statistics, (4) that did not report the association between vitamin B supplement and esophageal cancer or (5) that did not report the prognoses of esophageal cancer patients. Based on these criteria, 36 articles were eliminated and the remianing 19 articles that included 2036 esophageal cancer cases and 7086 controls were included for the meta-analysis [9–27].

### Characteristics and quality of include studies

The main characteristics of the 19 selected studies were outlined in Table 1. The studies were conducted in Asia, Europe, America, and Australia and were published between 1988 and 2016. In terms of the study design, 1 was a cohort study [15] and 18 were case-control studies [9–26]. Fifteen studies investigated dietary folate intake from food [10–13, 15–17, 20–27], and 4 studies examined serum folate levels in collected blood samples [9, 14, 17, 18].

**Table 1:** Characteristics of studies on folate intake and esophageal cancer risk

Author, year	Source of control	Study design	country	cancer type	measurement	OR(95%CI) for highest vs. lowest category	Participants (cases)	adjust	New Castle-Ottawa scale
Huang, 2013	PB	case-control	China	ESCC	Plasma	0.11 (0.04-0.33)	48 (6)	age, gender, smoking status, drinking.	8
Sharp, 2013	PB	case-control	Ireland	EAC	Dietary	0.52 (0.30-0.89)	136 (55)	age, gender, total energy.	8
Zhao, 2011	HB	case-control	China	ESCC	Dietary	0.61 (0.36-1.07)	174 (52)	age, gender.	6
Jessri, 2011	HB	case-control	Iran	ESCC	Dietary	0.08 (0.02-0.90)	144 (48)	age, gender, energy, BMI, smoking status, physical activity, education level, gastroesophageal reflux disease symptoms.	8
Chang, 2015	PB	case-control	China	Esophageal cancer	Plasma	1.58 (0.95-2.64)	178 (75)	age, gender, BMI, education, smoking status, alcohol drinking frequency.	8
Ibiebele, 2011	PB	case-control	Australian	EAC	Dietary	0.72 (0.53-0.98)	491 (117)	age, gender, education, BMI, alcohol intake, smoking status, energy intake, NSAID use.	8
Ibiebele, 2011	PB	case-control	Australian	ESCC	Dietary	0.78 (0.51-1.19)	430 (56)	age, gender, education, BMI, alcohol intake, smoking status, energy intake, NSAID use.	8
Aune, 2011	HB	case-control	Uruguay	Esophageal cancer	Dietary	0.29 (0.14-0.60)	2102 (70)	age, gender, residence, education, income, interviewer, smoking status, alcohol, dietary fiber, iron, BMI, energy intake.	7
Mayne, 2001	PB	case-control	America	EAC	Dietary	0.48 (0.36-0.66)	969 (282)	age, gender, site, race, proxy status, income, education, BMI, smoking status, alcohol, energy intake.	8
Mayne, 2001	PB	case-control	America	ESCC	Dietary	0.58 (0.39-0.86)	893 (206)	age, gender, site, race, proxy status, income, education, BMI, smoking status, alcohol, energy intake.	8
Bao, 2013	PB	case-control	China	ESCC	Plasma	0.43 (0.29-0.62)	212 (106)	age, gender, site.	7
Fanidi, 2014	PB	Nested case-control	European	ESCC	Plasma	1.03 (0.47-2.24)	255 (126)	age, sex, country, educational attainment, smoking status, alcohol intake.	8
Fanidi, 2014	PB	Nested case-control	European	EAC	Plasma	1.68 (0.79-3.56)	274 (26)	age, sex, country, educational attainment, smoking status, alcohol intake.	8
Galeone, 2006	HB	case-control	Italy and Swiss	ESCC	Dietary	0.68 (0.46-1.00)	404 (90)	age, center, education, BMI, smoking, alcohol drinking	7
Tavani, 2012	HB	case-control	Italy	Esophageal cancer	Dietary	0.26 (0.14-0.48)	443 (128)	age, gender, study center, year of interview, education, alcohol drinking, tobacco smoking, BMI, energy intake, physical activity.	6
Zhang, 1997	HB	case-control	America	EAC	Dietary	0.70 (0.30-1.70)	49 (18)	NR	6
Qin, 2008	HB and PB	case-control	China	Esophageal cancer	Dietary	0.52 (0.33-0.82)	360 (120)	NR	5
Brown, 1988	HB	case-control	America	Esophageal cancer	Dietary	0.70 (0.40-1.30)	629 (207)	Smoking status, alcohol intake.	6
Chen, 2009	PB	case-control	America	EAC	Dietary	0.50 (0.30-1.00)	573 (124)	age, gender, respondent type, BMI, alcohol intake, tobacco use, education level, family history, vitamin supplement use.	8
Yang, 2005	HB	case-control	Japan	Esophageal cancer	Dietary	0.77 (0.45-1.31)	270 (62)	Smoking status, alcohol intake, total energy.	6
Bollschweiler, 2002	PB	case-control	Germany	ESCC	Dietary	3.20 (1.30-9.10)	29 (16)	NR	6
Bollschweiler, 2002	PB	case-control	Germany	EAC	Dietary	5.00 (2.10-13.60)	38 (25)	NR	6

Abbreviations: PB, population-based; HB, hospital-based; NR, not reported; EAC, esophageal adenocarcinoma; ESCC, esophageal squamous cell cancer; OR, odds ratio;

CI, confidence interval; N/A, not available.

The quality of these studies was assessed by using NOS scale. The overall methodological quality of the studies is summarized in Table 2. Eleven studies had a score of 8 [9, 10, 13, 14, 15, 17, 19, 25], four studies had a score of 7 [16, 18, 20, 27] and the remaining six studies had a score of 6 [11, 12, 21, 22, 24, 26].

**Table 2:** Subgroup analysis of folate intake and risk of esophageal cancer

Cancer sites	Group	No. of Studies	OR(95%CI)	*P* for test	Heterogeneity test I^2^ (%)	*P*
Dietary folate intake		18	0.627 (0.557-0.706)	0.000	0.702	0.000
	Geographic locations					
	Europe	6	0.675 (0.522-0.873)	0.003	88.200	0.000
	Asia	3	0.610 (0.354-1.052)	0.001	0.000	0.548
	Australia	2	0.740(0.577-0.949)	0.018	0.000	0.749
	America	7	1.070(0.590-1.940)	0.000	37.800	0.140
	Dietary assessment					
	Validated FFQ/DHQ	8	0.623(0.527-0.738)	0.000	55.900	0.026
	N/A FFQ/DHQ	10	0.631(0.535-0.744)	0.000	78.100	0.000
	Histological type					
	NR	5	0.497(0.387-0.640)	0.000	61.000	0.036
	ESCC	7	0.726(0.597-0.883)	0.001	65.900	0.007
	EAC	6	0.623(0.519-0.748)	0.000	79.000	0.000
	Source of control					
	Hospital-based	8	0.556(0.450-0.686)	0.000	57.200	0.022
	Population-based	9	0.680(0.586-0.790)	0.000	78.800	0.000
	HB and PB	1	0.520(0.330-0.820)	N/A	N/A	N/A
	Study quality					
	Score≥7	10	0.603(0.525-0.694)	0.000	49.200	0.039
	Score<7	18	0.689(0.554-0.859)	0.001	81.700	0.000
Serum folate levels		5	0.709 (0.548-0.917)	0.009	0.883	0.000
	Country					
	Europe	2	1.327(0.772-2.282)	0.306	0.000	0.377
	Asia	3	0.519(0.441-0.791)	0.000	92.500	0.000
	Histological type					
	NR	1	1.580(0.948-2.634)	0.000	82.200	0.004
	ESCC	2	0.438(0.317-0.605)	N/A	N/A	N/A
	EAC	1	1.680(0.791-3.566)	N/A	N/A	N/A

Abbreviations: PB, population-based; HB, hospital-based; NR, not reported; EAC, esophageal adenocarcinoma; ESCC, esophageal squamous cell cancer;

OR, odds ratio; CI, confidence interval; N/A, not available, FFQ: food frequency questionnaire, DHQ: dietary history questionnaire, N/A: not available.

### Dietary folate intake

**Figure 2:**
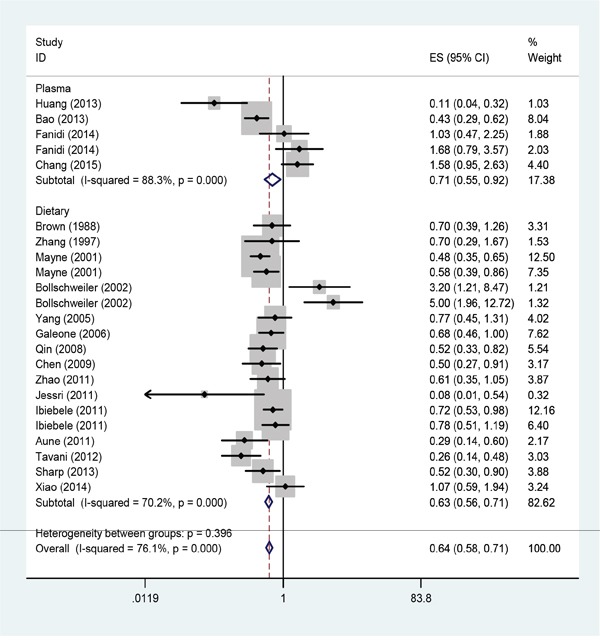
Forest plot between highest vs. lowest categories of folate intake and esophageal cancer risk

The link between dietary folate intake and esophageal cancer risk was analyzed in 14 case-control studies and 1 cohort study. The dietary folate intake data from the analyzed studies showed significant heterogeneity (I^2^=70.2%; *P*<0.001) and the pooled OR of esophageal cancer for the highest vs. lowest level of dietary folate intake was 0.63 (95% CI: 0.56-0.71; Figure 2).

Table 2 shows the associations between dietary folate intake and esophageal cancer risk in subgroup meta-analyses stratified based on geographic locations, number of studies, dietary assessment, histological type, source of controls (population-based or hospital-based) and study quality. With the exception of Asia and America, our data suggested an inverse association between dietary folate intake and esophageal cancer for all the analyzed sub-group strata.

### Serum folate level

As shown in Figure 2, the pooled OR for esophageal cancer in the highest vs. the lowest category of blood folate levels was 0.71 (95% CI=0.55-0.92) with significant heterogeneity in the analyzed data (I^2^ =88.3%; *P*<0.001). Because only 4 studies reported serum folate levels, subgroup analysis based only on the geographic locations and histological types were performed for those (Table 2).

### Dose-response meta-analysis

**Figure 3:**
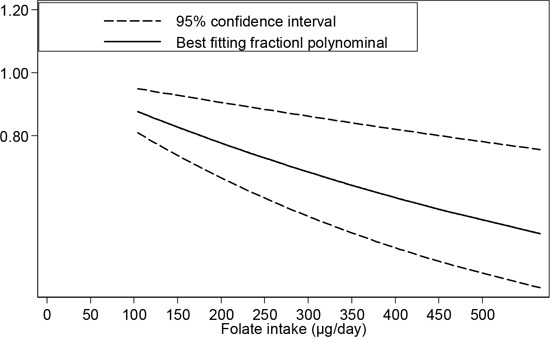
Dose-response relationship between folate intake and esophageal cancer risk

To study the relationship between dietary folate intake and the risk of esophageal cancer, dose-response of six case-control studies and one cohort study was analyzed. As shown in Figure 3, we observed a 12% decrease in the risk of esophageal cancer when the folate intake was increased to 100 μg/day (OR=0.88, 95%CI=0.83-0.95, *P*_linearity_=0.00).

Further, four case-control studies were analyzed to find the dose-response relationship between the serum folate concentration and the risk of esophageal cancer. The results of both the linearity test (*P*=0.29) and nonlinearity test (*P*=0.99) indicated that there is no linear or nonlinear relationship between the serum folate level and esophageal cancer risk.

### Publication bias

**Figure 4:**
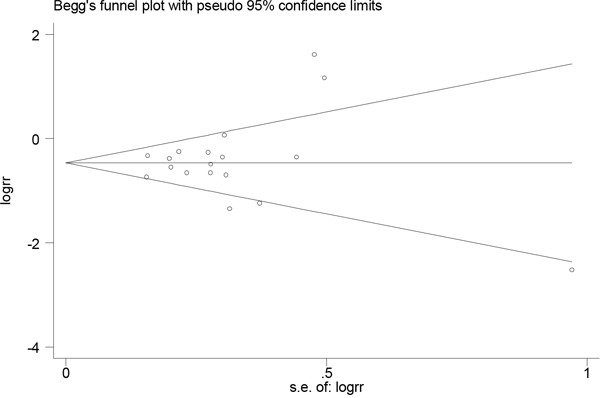
Funnel plot for assessing publication bias for folate intake and esophageal cancer risk

**Figure 5:**
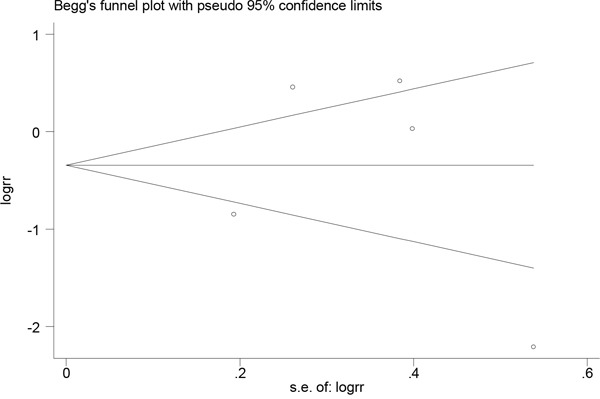
Funnel plot for assessing publication bias for serum folate level and esophageal cancer risk

Publication bias was evaluated through visual inspection of funnel plots (Figures 4 and 5). The results from the Egger and Begg tests revealed no evidence of publication bias (Egger: *P*=0.58 for dietary folate intake and *P*=0.95 for serum folate levels; Begg: *P*=0.94 for dietary folate intake and *P*=0.46 for serum folate levels).

### Sensitivity analysis of heterogeneity

**Figure 6:**
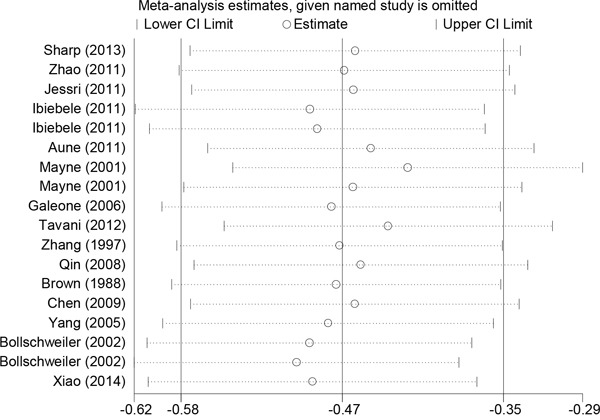
Sensitivity analysis of folate intake and esophageal cancer risk

When a sensitivity analysis of dietary folate intake was conducted, entailing sequential exclusion of individual studies from the pooled analysis, the original conclusion remained unaffected (Figure 6). Sensitivity analysis revealed that the key contributors to the heterogeneity among the results of the serum folate levels were data from two studies conducted by Chang *et al*. and Bao *et al*. Excluding those studies reduced the heterogeneity, and the pooled OR of 0.542 (95% CI:0.329-0.92) was similar to the main finding.

## DISCUSSION

As of December 2014, 82 countries worldwide have passed legislations mandating folic acid fortification in at least one industrially milled cereal grain [28]. Although there are many studies suggesting that folate exerts a protective effect against cancer development [29–31], recent conflicting reports suggesting increased risk of cancer related to folic acid fortification [32, 33] has necessitated further research into the roles of folate and other related micronutrients in cancer prevention. At present, there were few review studies investigating the association between folate level and esophageal cancer [30, 35, 36]. And The World Cancer Research Fund reported that folate protects against esophageal cancer are limitation of evidence [35]. In this study, we aimed to evaluate the role of dietary and plasma folate levels in determining the risk of esophageal cancer, and to characterize the dose-response relationship between esophageal cancer and folate intake.

This meta-analysis, which included 19 studies, provides a quantitative estimate of the association of dietary folate intake and serum folate levels with esophageal cancer risk. Our analysis found that the dietary folate intake and the serum folate levels are inversely associated with the risk of esophageal cancer. Moreover, the dose-response analysis demonstrated that dietary folate intake was linearly associated with the risk of esophageal cancer. A 12% decrease in esophageal cancer risk was recorded for a 100 μg/day incremental increase in folate intake.

The inverse relationship between dietary folate intake and esophageal cancer demonstrate in this study is consistent with a previous meta-analysis, which included nine case-control studies (OR=0.59, 95%CI=0.51-0.69). However, our study is more reliable as it included a larger number of participants and derived stronger conclusions regarding the association between dietary folate intake or serum folate levels and esophageal cancer risk. Furthermore, our study is the first comprehensive meta-analysis of dose-response that quantitates the association between dietary folate intake and the risk of esophageal cancer.

Since folate is a key modulator of DNA synthesis, repair and methylation, it was hypothesized to reduce cancer risk. Humans are unable to biosynthesize folate *de novo*, so they derive all the necessary folate from their diet [36]. Folate can be obtained from natural food sources, including citrus fruits, green leafy vegetables, cruciferous vegetables, legumes and cereals [3]. Since the first study [24] explored the association between folate and esophageal cancer in 1988, many epidemiological studies have investigated the relationship between folate intake and esophageal cancer risk. Evidence has suggested that low-folate status may contribute to carcinogenesis through complete conversion of dUMP via two mechanisms: (1) by conversion into dTMP, leading to uracil misincorporation into DNA, which could result in chromosomal breaks and mutations; and/or (2) by causing alterations in DNA methylation, which could in turn alter expression of proto-oncogenes and tumor suppressor genes [37]. However, recent reports have suggested that the effects of folate on carcinogenesis could depend on the dosage and the time of exposure [39]. Animal experiments demonstrated that whereas folate deficiency promoted carcinogenesis in normal cells, its supplementation could promote cancer progression in established pre-neoplastic lesions [39, 40].

In the subgroup analyses based on dietary assessment, histological type, source of control and study quality, we observed an inverse association between folate intake and esophageal cancer risk in all subgroups. However, in a sub-group analysis based on geographic locations, similar associations were absent in the Asian and American populations. The incidence and mortality rates of esophageal cancer showed wide geographic variation and there were marked differences between the high- and low-risk areas. People living in high esophageal cancer risk regions had significantly lower folate blood concentrations and nutrient intake than did those living in low risk regions [41]. This may explain why populations from different geographic locations have different results. The difference may also reflect complexity due to the presence of folate from various food sources, the occurrence of large amounts of folate in nature, and the diversity of dietary culture. The amounts of folate found in vegetables and fruits will depend on the type of cultivation, crop variety and location, as well as the specific morphological part of the plant eaten. Moreover, cultural differences in the storage and preparation of foods, particularly vegetables, likely affect this result [42]. In addition, the different study populations may vary in the ranges of folate intake and supplementation that could affect the relative impacts on esophageal cancer. We therefore performed a dose-response analysis to assess the relationship between folic acid intake and esophageal cancer.

Although our study is the first comprehensive meta-analysis to evaluate serum folate levels and the risk of esophageal cancer, there were only four studies that could be analyzed. This may have limited the precision of the estimations of associations and reduced the credibility of the results. A larger-scale study with greater statistical power will be needed to better assess the associations, particularly for the subgroup analyses of interactions.

Our study had several strengths that need to be highlighted. First, since our data included a broad folate intake range, it enabled accurate statistical analysis of the dose-response relationship between folate intake and the risk of esophageal cancer. Second, we assessed the methodological quality of the included studies by the Newcastle-Ottawa Scale (NOS). Third, our analysis provided an accurate assessment of the effect of folate levels upon esophageal cancer risk as we conducted subgroup analyses in specific populations. Further, high dietary folate intake and folate-rich foods may reflect a healthy lifestyle that includes other factors like never smoking, lower alcohol consumption and lower body weight that have been associated with a decreased risk of esophageal cancer. Therefore, factors such as age, sex, energy intake, alcohol use, smoking status, and treatment received were adjusted for in most of the studies included in this meta-analysis. Finally, sensitivity analyses of dietary folate studies showed that our results were robust, with no statistical evidence of publication bias.

The limitations of our study were as follows: (1) since our search yielded only one prospective study, our results could be affected by recall bias and selection bias; (2) none of the studies analyzed total folate intake, comprising folate from the diet along with folate from supplements; (3) the analysis used pooled data (individual data were not available), which prevented us from performing a more detailed analysis and obtaining more precise results.

In conclusion, the results of this meta-analysis suggest that greater dietary intake of folate and higher serum folate levels are protective against esophageal cancer. And the dose-response analysis of dietary intake of folate show that every 100 μg/day increase of folate can reduced 12% risk of esophageal cancer. Our analysis also indicates that in future, well-designed large prospective cohort studies that repeatedly measure folate intake with long follow-up periods and adjustments for all potential confounders are necessary to accurately verify the association of dietary folate intake and serum folate levels with esophageal cancer.

## MATERIALS AND METHODS

### Search strategy

Relevant articles were identified by two reviewers through systematic searches of the Medline, Pubmed, and Cochrane Library electronic databases (from database inception to Jul 2016). The search was performed using the terms (“folate” OR “folic acid”) AND (“cancer” OR “neoplasm” OR “carcinoma”) AND (“cohort study” OR “case-control studies”). In addition, we scrutinized references from relevant original reports, review articles and meta-analyses to identify other pertinent studies. No language restrictions were imposed.

### Study selection

A study was eligible for inclusion if the following criteria were met: (1) the study designed as a cohort, nested case-control or case-control study; (2) the study investigated the association between esophageal cancer and folate intake; and (3) the authors reported effect estimates (risk ratio [RR], or odds ratio [OR]) and 95% confidence intervals (CIs) for comparisons between high and low dietary folate intake or serum folate levels. When multiple levels of folate intake were presented, the ratio comparing the highest intake versus the lowest intake was chosen. In the case of duplicate studies, the most recently published study was chosen for inclusion.

### Data extraction and quality assessment

Data were extracted independently by two authors using a standard extraction form. The following data were extracted from each publication: the first author’s name, publication year, study design, geographic locations where the study was performed, type of controls in case-control studies, sample size (cases and controls or cohort size), measure and range of exposure, lowest folate level, highest folate level, difference between the highest and lowest folate levels, variables adjusted for in the analysis, and risk estimates with corresponding 95% CIs for the highest vs. lowest categories of folate intake or for each category. For studies that reported several multivariable adjusted-effect estimates, we selected the effect estimate that had been maximally adjusted for potential confounders.

Study quality was assessed using the Newcastle-Ottawa quality assessment scale (NOS), which is a comprehensive tool that has been validated for evaluating the quality of observational studies in meta-analyses [43, 44]. A NOS based on the following 3 subscales awarded a maximum of 9 points: selection of participants and measurement of exposure (4 items), comparability (2 items), and evaluation of methodological quality outcome (3 items). Studies with a score of 7 or higher were considered to be high quality [45, 46].

### Statistical analysis

We examined the association of folate intake and serum folate levels with the risk of esophageal cancer on the basis of the effect estimates (RR or OR) and 95% CI reported in each study. Heterogeneity was assessed using the I^2^ statistic, which is the proportion of total estimate variation attributable to study heterogeneity; I^2^ values of 25%, 50% and75% were used as cut-off points for low, moderate and high degrees of heterogeneity, respectively [47, 48]. We used a fixed effect model (Mantel-Haenszel method) when heterogeneity was negligible, and a random effect model (Dersimonian and Laird method) when heterogeneity was significant. We also performed a sensitivity analysis by removing individual studies from the meta-analysis when statistically significantly heterogeneity was detected. Several methods were used to assess potential publication bias. Visual inspections of funnel plots for esophageal cancer were conducted. The Egger and Begg tests were also used to statistically assess publication bias for esophageal cancer [49, 50]. We also conducted analyses stratified by study location, Histological type, Source of control, dietary assessment measures and Study quality.

Lastly, we conducted a dose response analysis using the median or mean folate level and the adjusted natural log of the RRs or ORs with their standard error (SE). When the folate intake was reported by range, we assigned the midpoint of the upper and lower boundaries in each category as the average intake. When the highest category was open-ended, we considered the width of the category to be the same as that of the adjacent category. When the lowest category was open-ended, the lowest boundary was set to zero [51, 52]. To derive the dose-response curve, we modeled folate using restricted cubic splines with four knots at the 5th, 35th, 65th and 95th percentiles of the distribution [53]. We included studies for this dose-response analysis only if they reported the distributions of cases and persons or person-years, as well as the ORs (RRs) and 95% CI with the variance estimates for at least three quantitative exposure categories [53, 54]. All tests were two sided with a significance level of 0.05. Statistical analyses were performed using STATA software (version 12.0; Stata Corporation, College Station, TX, USA).
